# Radiotherapy as a Backbone for Novel Concepts in Cancer Immunotherapy

**DOI:** 10.3390/cancers12010079

**Published:** 2019-12-29

**Authors:** Julijan Kabiljo, Felix Harpain, Sebastian Carotta, Michael Bergmann

**Affiliations:** 1 Department of Surgery, Division of General Surgery, Medical University of Vienna, Waehringer Guertel 18–20, 1090 Vienna, Austria; julijan.kabiljo@meduniwien.ac.at; 2 Comprehensive Cancer Center, Medical University of Vienna, 1120 Vienna, Austria; 3 Department of Cancer Cell Signaling, Boehringer Ingelheim RCV GmBH & Co KG., Dr. Boehringer Gasse 5–11, 1120 Vienna, Austria; sebastian.carotta@boehringer-ingelheim.com; 4 Ludwig Boltzmann Institute Applied Diagnostics, Medical University of Vienna, Waehringer Guertel 18–20, 1090 Vienna, Austria

**Keywords:** radiation, irradiation, immunotherapy, immune checkpoint inhibitors, PD-1, CTLA-4, STING, TMEM173, clinical

## Abstract

Radiation-induced immunogenic cell death has been described to contribute to the efficacy of external beam radiotherapy in local treatment of solid tumors. It is well established that radiation therapy can induce immunogenic cell death in cancer cells under certain conditions. Initial clinical studies combining radiotherapy with immunotherapies suggest a synergistic potential of this approach. Improving our understanding of how radiation reconditions the tumor immune microenvironment should pave the way for designing rational and robust combinations with immunotherapeutic drugs that enhance both local and systemic anti-cancer immune effects. In this review, we summarize irradiation-induced types of immunogenic cell death and their effects on the tumor microenvironment. We discuss preclinical insights on mechanisms and benefits of combining radiotherapy with immunotherapy, focusing on immune checkpoint inhibitors. In addition, we elaborate how these observations were translated into clinical studies and which parameters may be optimized to achieve best results in future clinical trials.

## 1. Introduction

Radiation has been a key therapeutic modality in the treatment of cancer since the beginning of modern oncology. The achieved reduction in tumor mass was historically attributed solely to its cytotoxic effects, as efficacy was usually restricted to the irradiated tumor. Nevertheless, some clinicians observed a rare therapeutic effect on distant metastases after irradiation of the primary tumor [1]. This distant tumor reduction has been deemed the abscopal effect, Greek for “off target”. The suggested contribution of the immune system to this finding [2] was confirmed by pre-clinical models [3,4]. Ablation of cytotoxic T-cells diminished the effect of 15 Gy external beam irradiation in mice [5]. Murine studies further indicated that irradiation also induced mediators of the innate immune axis such as type I interferons (IFNs). This cytokine was critical for the therapeutic potential of irradiation at the primary site [6]. Indeed, clinical trials adding recombinant IFNs to radiation have demonstrated some synergistic efficacy. Despite the fact that interferon-associated on-target toxicity limited the success of this combination [7,8], these attempts still clearly indicated that radiotherapy (RT) has immune-stimulating potential. In the light of novel immune-modulating drugs, which are currently being developed to counteract tumor-associated immunosuppression [9], the immunogenic “side effects” of irradiation have gained interest. It is hypothesized that irradiation could become a relevant player in tumor immunology when combined with such drugs. In this review, we delineate the immunological consequences of external beam radiation therapy and their clinical implications. Specifically, we discuss which types of cell death cancer cells succumb to after radiation and the immunomodulatory effects of respective modes of death. We furthermore elucidate the effects of radiation on cancer-associated immune cells and the tumor microenvironment. We give a detailed account of preclinical studies and their implications for the optimization of future clinical trials. Finally, we summarize recent clinical data gathered so far concerning radio- and immunotherapeutic combinations in various cancer types. It should be noted that, here, we do not discuss internal radiotherapy using radioactive isotopes, as currently there is little literature about its combination with novel immune-modulating drugs.

## 2. Radiation-Induced Immunogenic Cell Death

**Figure 1:**
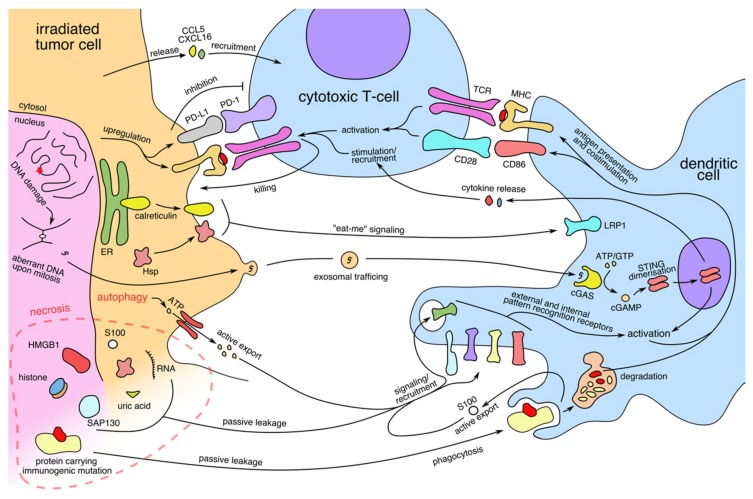
Immunomodulatory consequences of irradiation of tumor cells on dendritic cells and cytotoxic T-cells. Irradiation induces DNA damage, autophagy, and necrosis in the irradiated cells. This leads to stimulation of dendritic cells by danger-associated molecular patterns (DAMPs) mediated by various pathways. Antigen presentation by dendritic cells leads to activation of cytotoxic T-cells which can be blocked by immune checkpoint molecules expressed by tumor cells.

Radiation can induce various forms of cell death including apoptosis [10], mitotic catastrophe [11], autophagic cell death [12], necroptosis [13], and necrosis [14,15,16]. The type of cell death induced is dependent on the genetic background of cancer cells, the tumor microenvironment, and the dose of radiation applied. Higher doses of radiation in the range of 30 Gy result in necroptosis and necrosis, while apoptosis is predominantly induced at lower doses of 5 Gy [14]. This is relevant for the development of combined irradiation and immunotherapy protocols, as apoptosis is regarded as immune-silent and has even been associated with immune-inhibitory effects in murine TSA breast cancer cell line xenograft models [17]. Controversially, apoptosis has been shown to have immune stimulating properties under certain conditions [18,19], stimulating danger-associated molecular pattern (DAMP) presentation on the cell surface of apoptotic bodies [20]. Specifically, radiation induces calreticulin exposure to the cell membrane [21,22,23]. Here, calreticulin acts as a DAMP and “eat-me signal” through engagement of the low-density lipoprotein receptor-related protein 1 (LRP1) on phagocytic cells (Figure 1). Surface expression of heat shock proteins, another class of DAMPs acting through toll-like receptors (TLRs) and LRP1, was also described upon radiation [24,25,26]. Similarly, autophagic cell death, necroptosis, and necrosis release DAMPs into the intercellular space, as well as cancer-specific antigens. Both can lead to strong immunogenic responses [15,16,27]. Danger-associated molecular patterns act as immune activating signals and stimulate dendritic cells (DCs) towards a T-cell activating phenotype by binding to pattern recognition receptors [28] (Figure 1). The types of cell death inducing an immunological response are generally summarized as immunogenic cell death which can lead to initiation or enhancement of an anti-tumor immune response [28]. Autophagy and autophagic cell death contribute to anti-tumor immunity [27]. They may induce active transport of intracellular adenosine triphosphate (ATP) to the extracellular space as a result of low doses (2–4 Gy) of radiation [29]. Here, ATP acts as a DAMP through P2X purinoceptor 7 (P2X7) signaling [30]. It is one of few actively secreted DAMPs involved in radiation. S100 proteins constitute another actively secreted group of DAMPs. They are specifically released by macrophages upon phagocytosis and act through the receptor for advanced glycation end products (RAGE), toll-like receptor 4 (TLR4), and CD147 [31]. They have not been extensively studied in connection to radiation, but some reports indicate their levels in patient serum increase upon radiotherapy [32]. Furthermore, radiation can induce facilitating factors of phagocytosis which may subsequently lead to enhanced S100 protein secretion. Necroptosis and necrosis, occurring at higher doses of radiation, can additionally release a larger variety of intracellular proteins acting as DAMPs. High-mobility group protein B1 (HMGB1) is one of the most studied examples [33]. It induces strong activation of immune cells through TLR2, TLR4, and RAGE. High-mobility group protein B1 has not only been reported to activate the immune system, but it is also known to promote growth in pancreatic cancer and colorectal cancer models [34,35]. In addition, HMGB1 may stimulate growth of immunosuppressive effector cells [36]. Furthermore, DAMPs released into the intercellular space upon necrotic events include histones which activate TLR2 and TLR4 [37] as well as histone deacetylase complex subunit SAP130 activating macrophage inducible Ca2+-dependent lectin receptor (MINCLE) [38,39], uric acid activating the NLRP3 inflammasome [40,41], and RNA activating TLR3 [42] (Figure 1). Novel danger signals released upon necrosis are consistently being described, including mitochondrial transcription factor A [43], cyclophilin A [44], and F-actin [45]. Their roles in radiotherapy still need to be explored.

Surface intercellular adhesion molecule-1 (ICAM-1) expression also increased upon irradiation of lymphatic and vascular epithelial cells as well as malignant cells [46,47,48]. This cell adhesion molecule is important for immune cell migration into the tumor [49] and has been shown to enhance anti-tumor immunity [50,51]. Vascular remodeling and subsequent enhanced immune cell infiltration was also reported to facilitate effects of radiotherapy in mice [52]. A further effect of radiotherapy was locally enhanced expression of an increased intracellular peptide pool on the surface of tumor cells via major histocompatibility complex class I molecules (MHC-I) [53]. These molecules present intracellular peptides generated from degraded proteins on the cell membrane, where they may be detected by T-cells and lead to cytotoxic effects if recognized as foreign. Finally, irradiation can induce cellular senescence, leading to proliferative arrest and secretion of specific cytokines and growth factors known as senescence-associated secretory phenotype (SASP) [54].

Irradiation of cancer cells induces DNA double strand breaks and chromosomal aberration [55]. Irradiation-associated DNA damage appears to be critical for the induction of an immunogenic cell death. This is mediated by the cytosolic DNA sensing pathway and is caused by leakage of aberrant pieces of DNA into the cytosol upon mitosis, a phenomenon described as micronucleus [56,57,58]. Since DNA is only present in the cytosol under pathological conditions, including infections with DNA viruses and DNA damage, mammalian cells have developed a cytosolic DNA sensor pathway. Cytosolic DNA is sensed through direct interaction with the cyclic GMP–AMP synthase (cGAS) [59,60]. Activation of cGAS leads to synthesis of cyclic di-nucleotides (CDNs) which activate the stimulator of interferon genes (STING) protein (Figure 1). This results in engagement of various transcription factors such as IRF3 and NFκB and, subsequently, upregulation of cytokine production, most prominently type I IFN. A murine B16 xenograft model revealed, that STING signaling was necessary for inducing adaptive immune responses against the tumor [61]. In this study, dendritic cells were responsible for major STING dependent production of IFN in the tumor microenvironment. The fact, that radiation-induced leakage of nuclear DNA into the cytoplasm and subsequent activation of cGAS/STING is a key element of radiotherapy has been uncovered in pre-clinical mouse studies [62,63]. The exact mechanism of how radiation leads to the activation of the cGAS/STING pathway in various cells is currently not completely understood. Several possible scenarios have been put forward following their description in mice: (a) direct sensing of cytoplasmic DNA in cGAS/STING expressing cancer cells leading to cancer derived secretion of pro-inflammatory cytokines such as type I IFN that activate tumor associated dendritic cells; (b) irradiated cancer cells produce CDNs which are then transferred to neighboring innate immune cells via tight gap junctions leading to DC activation; (c) exosomal shedding of tumor-derived DNA to target dendritic cells [64]. However, a recent report highlighted the fact that a crucial parameter that determines whether radiotherapy induces cGAS/STING-mediated immunogenic effects is the dose of radiation applied [45]. High radiation doses not only have negative effects on the survival of immune cells in the tumor micro-environment but can also upregulate negative regulators of the DNA sensor pathway such as the three prime repair exonuclease 1 (Trex1) which degrades cytoplasmic nucleic acids and thereby reduces cGAS signaling [63]. This effect was shown for single doses of 20 Gy, while fractionated doses of 3 × 8 Gy seemed to induce sufficient cGAS activation and treatment response.

## 3. Effects of Radiation on the Tumor Immune Microenvironment

Tumor-infiltrating immune cells are essential in cancer pathogenesis and therapy. Understanding the effects of irradiation on the innate and adaptive parts of the immune microenvironment is crucial for designing potent combinations of radiation and immunotherapeutic agents.

### 3.1. The Adaptive Immune System

Early studies in mice indicate that cytotoxic T-cells are highly relevant for any immune-modulatory therapy even with agents such as bacterial endotoxin [65,66]. The dominant prognostic role of T-cells in human cancer was subsequently demonstrated by the group of Galon [67], showing that cytotoxic T-cells predicted disease-free and overall survival of patients with colorectal cancer. More recently, clinical and preclinical studies suggest that radio-therapeutic effects depend on cytotoxic T-cells as well [5,52,68]. T-cell attracting chemokine ligands induced upon irradiation are also important for the effects of radiotherapy, and their downregulation and subsequent loss of T-cell infiltration are described as a mechanism of radio-resistance in murine tumors [69,70]. Conversely, depletion of adaptive effector cells has been postulated as a concern in radiation therapy. In a recent report, Arina et al. [71] suggested the existence of highly radio-resistant CD8 positive cytotoxic T-cells in the tumor microenvironment, resisting both multiple low dose (5 Gy) and a single high dose (20 Gy) of radiation in a murine model. Even after radiation, these cells retained activity and motility. These results seem to corroborate reports of pre-existing immunity being necessary for potent immune responses to radiation, suggesting that pre-therapeutic T-cell presence may drive major anti-tumor effects upon irradiation [72]. Conversely, Chen et al. [73] reported a significantly inhibited cytotoxic T-cell responses after a single 20 Gy dose of radiation in a similar murine model. In this model, type I interferons were secreted upon irradiation which led to autocrine stimulation of the tumors via interferon alpha (IFN-α) receptors and Serpin B9 signaling, leading to immune-inhibitory reactions. Further evidence of opposing functions of interferons in an immune checkpoint blockade treatment setting was elucidated by Benci et al. [74]. This study showed that blockade of IFN-γ was able to either aid or inhibit checkpoint inhibitor-mediated therapy depending on the cell line used to generate the tumors in a murine model as well as exploring this effect in the context of radiation as a stimulator of interferon release. Furthermore, IFN-γ leads to upregulation of the programmed death-ligand 1 (PD-L1) protein [75,76]. Programmed death-ligand 1 is a ligand of the programmed cell death protein 1 (PD-1) immune checkpoint, a major immune-inhibitory signal which is often utilized by tumors to escape T-cell-mediated cytotoxicity. This indicates the importance of PD-L1 in radiotherapy, since irradiation leads to tumor cell IFN release [77]. Indeed, murine studies demonstrated PD-L1 upregulation upon irradiation and paved the way for rational combination of radiation and PD-1 axis blockade, showing synergistic effects in a murine cancer model [78]. This points to a two-sided effect of irradiation. Radiation exerts both immune-stimulatory and immune-inhibitory effects on cytotoxic T-cells within the tumor microenvironment. Further research into mechanistic determinants will be needed in order to maximize T-cell activity upon irradiation.

Intratumoral regulatory T-cells (T-reg) are a sub-type of immunosuppressive T-cells. They negatively regulate the effector functions of intratumoral CD8+ cytotoxic T-cells promoting tumor progression and growth. Whole-body irradiation of mice highly increased the ratio of peripheral T-regs towards other T-cell populations [79]. Radiation enhances intratumoral T-reg abundance in mouse models [80,81]. Interestingly, T-regs have been shown to better resist radiation-induced cell death than other T-cells [82]. In conclusion, irradiation may have unfavorable effects on intratumoral T-reg abundance. Therefore, radiation therapy may specifically benefit from the combination with T-reg depleting agents.

Even though the role of B-cells in cancer is less investigated than that of T-cells, a few studies suggest beneficial roles of B-cells in distinct types of cancer [83,84]. Both IgG and IgM antibodies were induced in patient samples of irradiated non-small cell lung cancer (NSCLC), hinting at adaptive processes involving B-cells being stimulated upon irradiation [85]. The presence of antibodies was not associated with improved survival though. The relevance of B-cells and the antibodies they generate is still poorly understood in the context of radiation therapy.

### 3.2. The Innate Immune System

Macrophages have been described as a major infiltrating immune cell population in a variety of cancers and often constitute either a major immunosuppressive or immune-activating factor in the tumor microenvironment, as they are considered key modulators of immune reactions [86]. It is thought that pro-inflammatory M1 macrophages are the first immunogenic effector cells that invade inflamed tissue and orchestrate pathogen clearance by other innate and adaptive immune cells. Upon clearance of pathogens, macrophages enter one of many immune-inhibitory and wound-healing promoting phenotypes, roughly summarized as M2. Tumors somewhat adapt to initiate the M2-like state to protect themselves. More recent analyses indicate that tumor associated macrophages (TAMs) are frequently activated macrophages which express both activating (M1) and suppressive (M2) surface molecules [87,88]. Irradiation appears to modulate TAMs toward a predominance of immune stimulatory molecules [51,89]. However, abundance of highly immunosuppressive macrophages upon irradiation has recently been described as well [90,91,92]. A murine study by Jones et al. [93] has recently observed that TAM may be polarized towards either suppressive or stimulatory phenotypes upon irradiation depending on the type of cancer. Reducing macrophage recruitment into the tumor microenvironment by colony-stimulating factor-1 (CSF-1) blocking antibodies [94] led to an increased abundance of M1 polarized macrophages in the tumor. This suggests that radiation consistently induces stimulatory phenotypes, yet highlights the capability of some tumors to swiftly re-establish an immunosuppressive environment. Conversely, the therapeutic effect of a CSF-R1 blocking antibody might be due to the reduction of myeloid-derived suppressor cells (MDSCs) in the tumor microenvironment [95]. Still, studies concerning distinct tumor types in patients are needed to evaluate the effects of irradiation on macrophages and set a time window of immuno-stimulation upon irradiation in order to optimally time potential combinations with other immunotherapeutic procedures.

The impact of MDSC on irradiation seems to be two sided [96]. They are rapidly generated in the bone marrow as a balancing response to various immune-stimulatory cytokines including HMGB1 [36], tumor necrosis factor alpha (TNFα) [97] or interleukin 1 beta (Il-1β) [98]. They are also induced by immunoinhibitory signals like indoleamine 2,3-dioxygenase (IDO) [99]. Since some of these signals, including HMGB1, increase upon irradiation, radiation can increase MDSC abundance. In this line, a clinical study in cervical cancer patients was able to show elevated circulating MDSC levels upon irradiation [100]. On the other hand, mouse models suggest high doses of irradiation (30 Gy) to decrease MDSC abundance in the tumor [101]. In combination with PD-1 axis inhibition, 12 Gy was enough to significantly reduce MDSC abundance within murine TUBO mammary cancer cell-line injection-based xenograft models [102]. In conclusion, MDSC abundance increases due to the radiation therapy. High doses of irradiation or a combination with checkpoint inhibitors may mitigate this effect.

Dendritic cells (DCs) are important antigen-presenting cells (APCs) in the context of adaptive tumor immunity. Dendritic cells have repeatedly been shown to be stimulated by danger signals released upon irradiation of cancer and stroma cells [15,103,104,105]. Tumor-associated dendritic cells take up cancer-specific antigens, migrate to draining lymph nodes, and cross-present these antigens to CD8+ cytotoxic T-cells which get activated, proliferate, and systemically lyse tumor cells expressing the respective cancer antigen. Deletion of DCs, termed basic leucine zipper ATF-like transcription factor 3 (BATF3) expressing cDC1, results in the loss of radiation-mediated induction of an adaptive T-cell immune response. One murine study indicated, that presence of DCs was as important as cytotoxic T-cell abundance for effects of radiotherapy [106]. These activated DCs can subsequently prime highly potent adaptive T-cell responses through antigen cross presentation. In vitro studies have shown the induction of an immunosuppressive phenotype of DCs upon irradiation [107,108]. In short, radiotherapy will, in most cases, enhance priming of adaptive immune responses through DC stimulation but can also induce a suppressive myeloid environment. A much better basic understanding of the determining key signals is needed.

Little is known about the effects of radiation on other tumor-associated immune cell populations including natural killer (NK) cells. Natural killer cells have recently become the focus of a variety of anti-tumor treatments, and interest in this cell population in the context of tumor immunity is rising [109]. Natural killer cells have been described to eliminate cells that lose expression of human leukocyte antigen (HLA) molecules and, therefore, the ability of presenting foreign proteins to adaptive immune cells on their cell surface [110]. Inhibitory signaling of HLA molecules towards NK cells has been proposed as a mechanism explaining this effect [110]. While doubt may arise as to whether HLA molecules upregulated upon irradiation may inhibit NK cell activity [53], there seems to be indications of stimulatory NKG2D ligands being simultaneously upregulated [111]. Intercellular adhesion molecule-1 (ICAM-1) surface expression was also induced upon irradiation to promote enhancing effects on NK cell cytotoxicity in human in vitro models [50]. Furthermore, chemokine (C-X-C motif) ligand 6 (CXCL6) was upregulated in a human in vitro model of irradiation, leading to enhanced NK cell migration [112]. Surface calreticulin expression, an endogenous danger signal expressed upon irradiation [21,22], was shown to enhance NK cell-mediated cytotoxicity [113]. In a further in vitro model, low-dose radiation has shown to induce proliferation and activate NK cells [114]. Animal studies combining radiotherapy with adoptive NK cell transfer revealed promising results, as they observed enhanced anti-tumor effectiveness of NK cells [115,116]. Stem cell-like cancer cells were successfully targeted in a mouse model of adoptive NK cell transfer in combination with radiotherapy [117]. These stem cell-like cells are thought to replenish tumor cell populations following various therapeutic approaches and to be especially hard to target while contributing to recurrences [118]. Similar to T-cells, some NK cells can exhibit immunoinhibitory properties. These regulatory NK cells have been shown to inhibit T-cell responses in viral infections and are suggested to have pro-tumorigenic roles in cancer [119,120]. A study involving radiotherapy and hyperthermia has shown a complex and time-dependent role of NK cells in radiation-induced anti-tumor immunity employing a B16 melanoma model [121]: one-time depletion of NK cells shortly before radiotherapy combined with hyperthermia may increase therapeutic effectiveness by reducing immunoinhibitory NK cell subsets. Nevertheless, one-time NK cell depletion during radiotherapy decreased its effectiveness, indicating necessity of NK cells in this setting.

## 4. Optimizing the Combination of Irradiation and Immune Checkpoint Inhibition—Bench to Bedside

A major achievement of immune-oncology was the clinical development of immune checkpoint inhibiting antibodies. This therapeutic strategy is based on blocking inhibitory receptors on immune cells which are used by cancers to shield themselves from immune responses. Important examples include the cytotoxic T-lymphocyte-associated protein 4 (CTLA-4) blocking antibody ipilimumab, the programmed cell death protein 1 (PD-1) blocking antibodies nivolumab and pembrolizumab as well as the programmed death-ligand 1 (PD-L1) blocking antibodies avelumab, atezolizumab, and durvalumab [122,123,124,125,126,127,128]. These drugs were able to induce significantly enhanced therapeutic benefits compared to conventional therapy and were approved for treatment of various malignancies [129]. Nevertheless, only a limited number of patients benefited from immune-checkpoint-blockade, and a widespread search for mechanisms, predictive markers to select eligible patients, and combination therapies for increasing the amounts of patients in remission after immunotherapy began. One major finding was the observation that the amount of cytotoxic CD8 positive effector T-cells infiltrating the tumor was a significant predictor of response to PD-1 inhibition [130]. Therefore, it was postulated that treatments enhancing infiltration of T-cells into the tumor microenvironment, like radiotherapy, may increase patient response rates to checkpoint inhibitor treatment. This led to renewed interest in the immunological effects of radiation therapy and research into synergism between irradiation of cancer and immunotherapy. An early study was able to demonstrate that external beam irradiation increases response rates to CTLA-4 checkpoint inhibition in a murine 4T1 cell line-based xenograft model [131]. The CTLA-4 blocking antibody ipilimumab has recently been demonstrated to deplete T-reg cells, which abundantly express CTLA-4, via antibody dependent cell-mediated cytotoxicity (ADCC) [132]. Since irradiation has been shown to enhance T-reg abundance [79,80,81,82], the synergy of radiotherapy and CTLA-4 blocking antibodies may, to some extent, be due to the T-reg depletion. Nevertheless, further preclinical models assessed PD-1 axis blockade in combination with radiotherapy as well, suggesting highly improved outcomes with this combination [78,102,133,134,135,136]. A murine model compared radiotherapy in combination with CTLA-4 blockade with a combination of dual CTLA-4 and PD-1 blockades. The results indicated a significant improvement of median survival in the dual checkpoint blockade group [137]. While proposing a potent combination of novel and well-established therapeutics, studies elucidating optimal therapeutic sequence and dosing are needed.

Recent reports suggested that timing of external beam radiation and checkpoint inhibition may be an exceedingly important factor to consider when combining these two treatment modalities [138]. The CTLA-4 inhibition was shown to act most potently when given seven days before external beam radiotherapy in a murine model CT26 cell line xenograft model of 20 Gy single dose irradiation, compared to treatment one or seven days after irradiation [139]. It was later observed that immune checkpoint expression was induced by radiotherapeutic intervention and following mouse experiments suggested potent synergy with blockade of the PD-1 axis [78]. Specifically, checkpoint blockade yielded best results applied at the onset of short course external beam irradiation (five times 5 Gy) with poorer results when administered 7 days after the last course of radiation. The observed differences in optimal timing of CTLA-4 and PD-1 axis blocking agents may stem from the distinct roles of these pathways in immune activation [140]. The immune-inhibitory protein CTLA-4 on T-cells binds to the co-stimulatory ligand glycoproteins B7-1 and B7-2 (CD80 and CD86) during cross presentation of foreign peptides and inhibits binding of these ligands to the co-stimulatory receptor CD28. In the context of cancer immunology, this missing CD28 stimulation can dampen the generation of active cytotoxic T-cells in tumor-associated draining lymph nodes [140]. Conversely, PD-1 directly inhibits cytotoxic T-cell responses. This effect is often utilized by cancer cells through expression of its ligand PD-L1, resulting in evasion of T-cell cytotoxicity [140]. Therefore, the observed optimal timing of CTLA-4 blocking agents several days before irradiation stems from their enhancement of T-cell induction in the lymph nodes, while PD-1 axis blockade works best when given concomitantly due to the fact of its action directly in the tumor microenvironment.

To compare different dosing schemes, a mouse model of CTLA-4 blockade and different external beam irradiation protocols was explored by Dewan et al. [141] in a 4T1-based breast cancer model. There was no significant effect of irradiation or CTLA-4 blockade alone in this specific model, yet combination was most effective when tumors were irradiated three times with 8 Gy, while five times 6 Gy radiation treatments were less effective. A single 20 Gy treatment showed the least synergy with the CTLA-4 blockade in controlling tumor growth. Furthermore, in a murine MC38 xenograft model, two strong doses of irradiation (=hypofractionated, 2 × 8 Gy) were significantly more effective at inducing anti-tumor immunity in combination with PD-1 inhibition than a slightly higher overall dose delivered in multiple small fractions (=hyperfractionated, 10 × 2 Gy) [142]. On the other hand, irradiation above certain threshold doses induced immunoinhibitory effects, which were mediated by TREX, an inhibitor of the STING/cGAS pathway [63]. In the latter study, fractionated 3 × 8 Gy external beam irradiation was significantly more effective in inducing immune responses via the STING pathway than a single 20 Gy dose. In patients, single doses above 10 Gy, where this effect may become relevant, are usually only applied when advanced machinery like stereotactic radiotherapy is used. While the clinical benefits of combined radio- and immunotherapy with high single doses of radiation (18–22 Gy) have been observed [143,144], fractionation at 3 × 9 Gy was more effective in controlling brain metastasis than single doses between 18 and 20 Gy [145]. Conversely, Twyman-Saint Victor et al. [137] showed sufficient synergy of checkpoint inhibition and a single 20 Gy dose of external beam irradiation in a murine B16 melanoma xenograft model, suggesting immunosuppressive thresholds and optimal fractionation of radiotherapy may differ between specific tumor backgrounds. Furthermore, specific immunoinhibitory cell populations were also reduced upon high dose irradiation (30 Gy) of murine CT26 and MC38 colon cancer models [101]. In a study involving three different cell line-based subcutaneous murine xenograft models, five doses of 2 Gy external beam irradiation seemed sufficient for potent synergy between PD-1 blockade and radiotherapy [78]. Overall, these studies suggest that for each separate tumor type there is an optimal dose of radiation for inducing immunity and combination with immune checkpoints. Elucidating exact thresholds for specific patient cohorts may help guide future radiation treatments and further enhance their effectiveness.

### Additional Considerations

A more novel and less anticipated aspect in radioimmunology is the finding that multiple site external beam irradiation shows superior immunological effectiveness compared to single site irradiation, especially in the context of combination therapy with immune checkpoint inhibitors [146]. It was postulated, that multiple metastatic sites may develop distinct tumor-associated-antigens and therefore adaptive immune responses limited to the irradiated site. The first hint towards this effect was shown in a small clinical cohort of various cancer types treated with checkpoint inhibition and radiation [147]. Here, different tumor localizations led to distinct therapeutic outcomes of combined radio and immunotherapy. Through further review of published data Brooks et al. revealed further clinical evidence, that multiple site irradiation may enhance efficacy of this combination [146].

Accidental irradiation of draining lymph nodes is a related consideration. A murine study using MC38 and B16 cell line xenograft models has recently implied that draining-lymph-node irradiation inhibits the induction of CD8 T-cell mediated immunity when checkpoint inhibition and radiotherapy are combined [148]. This is corroborated by the observation, that intratumoral T-cells of murine tumors withstand radiation much more effectively than T-cells in healthy tissue [71]. These results are of particular interest to radiation oncologists designing combined radio- and immunotherapeutic interventions. Patient outcomes may be improved by excluding lymph nodes from the field of irradiation in case such strategies are applied.

Mathematical models may help determine optimal dosing schemes for combined radio- and immunotherapy. Utilizing murine TSA breast cancer xenograft models Serre et al. have proposed a mathematical model for predicting optimal radiation dose and fractionation for maximizing immunological effects of radiation and combinations with immunotherapy [149]. This model might aid in design of future preclinical and clinical studies involving such combinations, and the development of similar models towards human clinical application may enhance patient outcome in the future.

Furthermore, one must take into consideration that most murine models exploring efficacy and mechanisms of combined radio- and immunotherapy utilize tumor models generated by injection of tissue-culture adapted cancer cell lines into mice. While yielding homogenous large tumors which are practical to handle in an experimental setting, the cell lines used are highly adapted to growth in cell culture flasks and may be genetically distinct from naturally occurring tumors [150]. While some effects discussed here were replicable in patients, utilizing transgenic murine cancer models as summarized by Day et al. [151] or primary human immune-organoid-cocultures [152] may enhance relevance of future preclinical experiments.

## 5. Current Clinical Insights on Irradiation and Immune Checkpoint Inhibitor Combination Therapy

**Figure 2:**
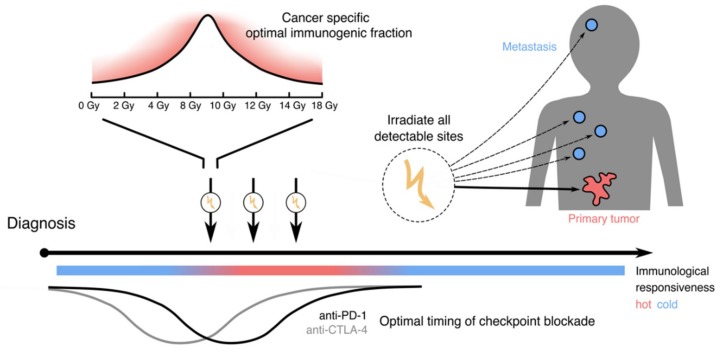
Combination of external beam radiotherapy and immune checkpoint inhibiting antibodies. The optimal timing of immune checkpoint inhibition appears to be in the early phase of radiotherapy. Preclinical models indicate that cytotoxic T-lymphocyte-associated protein 4 (CTLA-4) blockade may be more effective when administered days before irradiation. The optimal dose of radiation as determined in mouse models is indicated. The red gradient indicates suggested variability in cancer-specific clinical settings. Preliminary clinical studies have reported improved outcomes when irradiating multiple sites.

These promising preclinical results prompted multiple trials and retrospective analyses towards combining external beam irradiation with checkpoint inhibition in a clinical setting. Retrospective analyses of checkpoint blockade treatments after radiation therapy showed encouraging effects of ipilimumab in melanoma brain metastasis patients [153,154,155,156,157] (Table 1). Two initial prospective clinical studies have combined ipilimumab with external beam radiotherapy in melanoma [68,137] (Table 2). These clinical cohorts lacked a control group receiving established ipilimumab treatment only. Nevertheless, response rates were higher in comparison with previous large cohorts treated with ipilimumab monotherapy [122] (Table 1). In one study, melanoma metastases were irradiated 2–3 times with 6 to 8 Gy followed by multiple injections of ipilimumab 3 to 5 days after the last course of radiotherapy [137]. The combination was well tolerated. Eighteen percent of the 22 patients achieved partial response, and 18% had a stable disease. Another study chose to perform irradiation concomitantly with ipilimumab, starting ipilimumab treatment slightly before performing a variety of irradiation schedules devised individually for each patient by the radiation oncologist [68]. Of the 22 patients enrolled, 15% archived complete remission at a median follow-up of 55 weeks, with a further 15% showing partial response. Those differences suggest that timing of checkpoint inhibition within the course of irradiation is important and suggest further clinical randomized studies to evaluate this issue. Yet preclinical models also suggest a benefit of concomitant treatment as compared to sequential application [78]. Inhibition of PD-1 was also recently assessed in combination with external beam irradiation for melanoma brain metastasis in four separate small retrospective patient cohorts [143,158,159,160,161] (Table 3). While patient benefit compared to conventional treatment was difficult to determine due to the small cohort sizes, a meta-analysis revealed improved survival of combined irradiation and either PD-1 axis or CTLA-4 blockade compared to irradiation alone [162]. A retrospective analysis of patients treated for brain metastases of malignant melanoma revealed that inhibition of the PD-1 axis was more effective than inhibition of CTLA-4 in combination with external beam radiotherapy and that concurrent dosing (at least 4 weeks within the two treatments) was necessary to induce best responses [163]. Independent retrospective studies and one meta-analysis confirmed these results [145,164,165,166,167,168,169,170,171] (Table 4), also showing the superiority of combining radiation with PD-1 inhibitors compared to combination with other agents such as v-Raf murine sarcoma viral oncogene homolog B1 (BRAF) or dual specificity mitogen-activated protein kinase kinase (MEK) inhibitors [172,173] (Table 4). One retrospective study showed longer overall survival of patients irradiated more than 16 weeks after initiation of ipilimumab, compared to patients irradiated within 16 weeks of starting ipilimumab treatment [174]. In general, these studies confirm the importance of the concomitant timing of external beam irradiation and checkpoint inhibition that were postulated by preclinical studies (Table 4, Figure 2) [78,138,139]. The impact of dose and fractionation of radiotherapy was similar in preclinical models as well (Table 2, Table 3 and Table 4, Figure 2) [144,145].

More recently, the combination of external beam radiation therapy and checkpoint inhibitors was tested in patients with thoracic malignancies. A retrospective study by von Reibnitz et al. [175] involved 79 patients with various cancer diagnoses, most commonly lung cancer and melanoma, and treated with either PD-1 axis or CTLA-4 blockade and irradiation of thoracic primary tumors or metastases. This study aimed to explore differences in toxicity between concomitant and sequential therapy and found no significant differences, confirming the feasibility of concomitant treatment as a therapeutic option. A prospective study was able to show prolonged progression-free survival in a cohort of 473 NSCLC patients treated with durvalumab after chemo-radiotherapy, compared to 236 patients treated with placebo after chemo-radiotherapy [176]. Another prospective study showed that NSCLC patients receiving pembrolizumab had longer progression-free survival if they had received radiotherapy before [177]. These two studies suggest that the effects of irradiation and PD-1 inhibition are non-redundant and synergistically enhance patient outcomes in NSCLC. Conversely, large-scale analysis within the National Cancer Database of the United States of America revealed no indications of synergy of external beam radiotherapy and checkpoint inhibition in NSCLC, showing an advantage of either checkpoint inhibition or stereotactic radiotherapy alone over conventional radiotherapeutic approaches [178]. A retrospective analysis of NSCLC metastasized to the brain revealed no significant differences in survival among patients treated with radiation with or without checkpoint inhibitors [179]. A single center retrospective analysis of NSCLC patients showed acceptable adverse reactions in combination therapy of radiotherapy and nivolumab [180]. No relevance of timing of nivolumab on patient outcome was reported in this study. On the other hand, a recent retrospective study hinted at enhanced survival of NSCLC patients which were previously treated using radiotherapy [181]. In conclusion, NSCLC prospective and retrospective studies show survival benefits after combined external beam radiation and checkpoint blockade, while, controversially, a meta-analysis predicted no such synergy (Table 5).

A study explored CTLA-4 blockade combined with external beam radiotherapy in a cohort of various cancers [147]. The small cohort size and variety of cancer backgrounds limits the value of this study. A small phase 1 study combining the novel PD-1 blocking protein AMP-224 with radiation and low-dose cyclophosphamide did not seem to prolong survival of colorectal cancer patients compared to historical controls [182]. Two case series of Hodgkin’s lymphoma patients refractory to conventional therapy were treated with external beam radiotherapy and checkpoint inhibition using either PD-1 or CTLA-4 blocking antibodies and showed more favorable outcomes than historically observed for the respective agents alone [183,184] (Table 6). Microsatellite stability has been described as a major negative prognostic factor for immunotherapeutic response [185]. In a case series comprising three microsatellite stable intrahepatic cholangiocarcinoma patients, external beam irradiation appeared to enhance treatment with PD-1 axis blockade [186]. This study gives the first indications, that radiation might reverse the negative prognostic effect of microsatellite stability and resulting low tumor mutational burden in checkpoint inhibitor treatment. A prospective study comparing external beam irradiation combined with ipilimumab to radiation only in prostate cancer-associated bone metastases found no significant difference among groups [187]. Further trials combining radiotherapy with checkpoint blockade are currently being initiated for patients with advanced stage head and neck squamous cell carcinoma, breast cancer, and mucosal melanoma [188,189,190,191]. Studies are still ongoing exploring the synergistic effects of radiation and checkpoint blockade in various malignancies [192].

Most clinical studies exploring external beam irradiation in combination with checkpoint inhibitors were conducted in a metastatic setting. However, recent data from murine xenograft models of a neoadjuvant treatment setting show promising effects of immunotherapy on survival in the latter yet non-metastatic setting [193]. Checkpoint inhibitor treatment alone exhibited favorable survival rates in neo-adjuvantly treated melanoma, NSCLC, and glioblastoma cohorts [194,195,196]. Potentially, a reservoir of antigen of the primary tumor may be needed for the induction of strong adaptive immune responses which then exerts its effect on distant micro-metastasis once the primary tumor is removed. Neo-adjuvantly applied combined radio- and immunotherapy might further potentiate such effects. In this line, three clinical trials exploring this combination in a neoadjuvant setting were started and are currently being conducted in sarcoma and colorectal cancer patients (i.e., NCT03463408, NCT04124601, [197]). This development marks a new frontier for combined radio- and immunotherapy.

Taken together, these reports demonstrate a potentially potent synergy of external beam radiation, checkpoint blockade, and surgery in various types of cancer and treatment settings. A recent review on safety and adverse effects also concluded that the current data point to these combinations being, overall, well tolerated in a clinical setting [198]. Therefore, radiation and immunotherapeutic agents, especially immune checkpoint inhibitors, constitute a potent and safe combination and clinical trials broadening the range of cancer types that can be treated with this combination are called for.

## 6. Novel Therapeutic Combinations

Although the combination of radiotherapy with immune checkpoint blockade has shown synergy and has been clinically explored, therapy responses vary widely. Crittenden et al. [72] have suggested that pre-existing immunity is necessary to enable potent anti-tumor effects of external beam radiation and checkpoint inhibition. This indicates that an additional agent might improve combined radio- and immunotherapy response rates and lead to higher rates of complete remissions. Multiple studies have recently implied that rational combinations of an additional immunologically active agent to radiation and checkpoint inhibition might increase effectiveness and response rates to these combined treatments.

Hammerich et al. [199] described an in situ vaccination strategy aided by radiotherapy and checkpoint inhibition. External beam irradiation was meant to damage cells and release neoantigen. This was followed by injection of a Toll-like receptor 3 (TLR3) agonist and a DC engager. This attracted DCs which, in turn, phagocytized neo-antigenic proteins. The TLR3 agonist activated the DCs which wandered into lymph nodes and cross presented neoantigen to T-cells, activating an adaptive immune response against the tumor and its metastases. Subsequent checkpoint inhibition was performed to overcome the tumor’s immunosuppressive environment and enable effector T-cells to attack tumor cells. This approach seemed to prime potent immunological responses in mice. The same combination, without PD-1 axis inhibition, resulted in regressions in tumor mass in a twelve-patient collective with indolent non-Hodgkin lymphoma, while the clinical trial is ongoing [199].

Low-dose cyclophosphamide has been postulated to block T-reg activity with some measure of selectivity [200]. A study used cyclophosphamide in combination with the inducible nitric oxide synthase (iNOS) inhibitor N6-(1-iminoethyl)-L-lysine (L-NIL) to enhance responses to external beam radiation and dual checkpoint inhibition [201]. This led to increased response rates compared with irradiation and checkpoint inhibition alone in two cell line injection-based murine tumor models. Cyclophosphamide and L-NIL increased T-cell as well as NK cell and dendritic cell infiltration. Macrophages and neutrophils, both capable of being pro- as well as anti-tumorigenic, were also elevated. Exhausted T-cells marked by increased PD-1 expression were induced by this combination, explaining reported synergy with checkpoint inhibitors. There seemed to be no effect of this combination on immunosuppressive T-regs. In a clinical study enrolling 15 patients with colorectal cancer, low-dose cyclophosphamide combined with a novel PD-1-blocking protein as well as two different courses of radiation was well tolerated but did not seem to increase survival rates in comparison to previous similar cohorts treated conventionally [182] (Table 6). The limiting factors of this study included that the examined patient collective was heavily pretreated, and the microsatellite status was largely unknown.

Tyrosine kinase inhibitors have been shown to promote the therapeutic effects of immune checkpoint inhibitors. In this line, Caetano et al. [202] explored the combination of an anti-PD-1 antibody with a MER proto-oncogene tyrosine kinase inhibitor (MerTKI) and three 12 Gy doses of radiation in a murine flank model of NSCLC and pancreatic cancer. Triple combination enhanced survival reduced irradiated tumor size as well as halting growth of the non-irradiated flank tumor which single treatments and dual radio-immuno-therapeutic combination did not achieve. The effects were dependent on CD8+ T-cells and NK cells, suggesting both adaptive and innate components and polarized macrophages to a more desirable phenotype [202].

Since indoleamine-pyrrole 2,3-dioxygenase (IDO1) upregulation was noted a major immunoinhibitory pathway upon combined radio- and immunotherapy in glioma patients, Ladomersky et al. [203] evaluated IDO1 inhibition with radiation and PD-1 inhibition [203,204]. In this GL261 glioma cell line xenograft model, only triple combination of irradiation, PD-1 inhibition, and IDO1 inhibition achieved durable responses. Radiation and PD-1 blockade were performed concomitantly, while IDO1 inhibition was resumed for multiple weeks.

Tumor-associated macrophages often constitute a major immunosuppressive cell lineage in the context of cancer [86]. In a murine xenograft model of irradiation, a PD-1 blockade combination with an induced colony-stimulating factor 1 (CSF-1) inhibitor significantly reduced external beam radiation-associated repopulation of the tumor microenvironment with macrophages and led to potent synergy due to the lack of immunosuppressive signaling from macrophages [93].

Two murine studies suggested triple combination of external beam irradiation, PD-1 blockade, and CD137 agonism to synergize in cancer treatment [205,206], suggesting mechanistic target of rapamycin (mTOR) signaling to be involved in the therapeutic mechanism. Since transforming growth factor beta (TGF-ß) upregulation was observed as a result of radiotherapy [207], Rodríguez-Ruiz et al. [208] explored TGF-ß inhibition combined with CD137 agonism to enhance synergy between irradiation and checkpoint blocking agents, showing promising results in mice.

Mouse experiments have shown that external beam irradiation more potently synergized with a combination of CTLA-4 and PD-1 blockade than with the respective single agents [137]. In a retrospective clinical analysis, a small (*n* = 4) group of patients treated with this combination exhibited higher one-year overall survival than groups treated with radiation and respective single agents [173]. A mouse study has shown the merit of blocking other immune checkpoints in combination with PD-1 axis blockade and radiation in order to maximize effectiveness [209]. Inhibiting the checkpoints lymphocyte-activation gene 3 (*LAG-3*) or T-cell immunoglobulin and mucin-domain containing-3 (TIM-3) in combination with PD-1 axis blockade and irradiation enhanced survival of melanoma cell line xenograft-bearing mice. The combination with a TIM-3 blocking antibody was further corroborated in human NSCLC cell line xenografts in mice [210]. Also, triple combination with a TIGIT blocking antibody showed promising results in a CT26 xenograft model [211].

Immunological effects have been demonstrated for some chemotherapeutic agents [212], yet the well-established chemotherapeutic cisplatin has been considered non-immunogenic so far. Recently, cisplatin was shown to potently enhance combined external beam irradiation and checkpoint blockade in multiple murine xenograft models [213]. Another murine study combined irradiation and checkpoint blockade with cisplatin and a CD137 agonist, also showing synergistic effects [214]. These preclinical studies suggest that some chemotherapeutic agents may prove synergistic in combined radio- and immunotherapy.

In 2015, the herpes simplex virus based oncolytic virus T-vec has received clinical approval and is now routinely applied by intratumoral injection in late-stage melanoma [215,216]. A mouse study utilizing a B16 melanoma xenograft model has shown synergy of T-vec and external beam radiotherapy, and prospective studies are being performed exploring this combination in melanoma and sarcoma (i.e., NCT02819843 and NCT02453191 [217]). Recently, a preclinical model evaluated an oncolytic Newcastle disease virus in combination with radiotherapy and PD-1 or CTLA-4 inhibition, showing synergistic local and abscopal responses [218]. A case report of combined T-vec, PD-1 inhibition, and radiation leading to complete CNS and partial systemic response has prompted a prospective clinical trial exploring similar combinations in triple-negative breast cancer and NSCLC (i.e., NCT03004183 [219]). This research indicates that combination of radiotherapy and checkpoint inhibitors with oncolytic viruses is a promising strategy to further expand the collective of patients responding to immunotherapy.

Taken together, these preclinical and clinical studies suggest that external beam radiation and checkpoint inhibition can be further enhanced by the addition of a third agent, moving clinical development closer to unprecedented response rates in the future of immune-oncology. But while we know that dual checkpoint blockade may also lead to potentiated efficacy, toxicity is increased in this combination [220]. Therefore, further studies into possible toxicities of such triple combinations of radio- and immunotherapy need to be conducted and the most advantageous combinations selected before they can become clinical reality.

## 7. Conclusions

In conclusion, the current clinical and preclinical data point toward external beam radiotherapy being a potent enhancer of checkpoint-inhibition-based immunotherapy due to the variety of immunomodulatory properties. First, the results from clinical trials combining these treatment modalities are promising. Here, we were able to summarize multiple previously observed factors that may still be optimized in order to maximize the effect of this treatment combination. These include optimizing timing and dose fractionation of the respective therapeutic agents, irradiating multiple sites, and adding a third immunomodulatory agent. Overall, these considerations may form the basis to increase patient response rates to the combination of irradiation and checkpoint inhibition.

## Figures and Tables

**Table 1:** Overview of clinical trials concerning combination of radiotherapy and cytotoxic T-lymphocyte-associated protein 4 (CTLA-4) blockade treatment in metastatic melanoma. Clinical trials are sorted primarily by cancer and treatment setting, secondary criteria being chronological. Short descriptions of patient collective, treatment, and outcome are included.

Trial	Timing of Checkpoint Inhibition	Prospective (Y/N)	Cohort Description	Treatment	Outcome
Anti-CTLA-4, Metastatic Melanoma					
Knisely et al. 2012 [153]	various	N	melanoma brain metastases	50 patients received various doses of radiotherapy (r), 27 patients received various doses of radiotherapy and ipilimumab before, during or after radiotherapy (i)	median overall survival was 4.1 months (r) and 21.3 months (i)
Silk et al. 2013 [156]	various	N	melanoma brain metastases	33 patients received radiation and ipilimumab (i), 37 patients received radiation only (r)	median overall survival was 5.3 months (r) and 18.3 months (i)
Barker et al. 2013 [174]	comparative	N	melanoma brain metastases	19 patients received radiotherapy in the first 16 weeks of ipilimumab treatment (e), 11 patients received radiotherapy more than 16 weeks after start of ipilimumab treatment (l)	median overall survival was 9 months (e) and 39 months (l)
Twyman-Saint Victor et al. 2014 [137]	after	Y	metastatic melanoma, stage M1, mostly M1c (68%)	22 patients received 2–3 × 6–8 Gy targeted radiotherapy and ipilimumab 3–5 days after the last irradiation	18% partial response, no complete remission
Kiess et al. 2015 [154]	comparative	N	melanoma brain metastases	16 patients received single fraction 15–24 Gy radiation, of these 15 received ipilimumab during (d), 12 before (b) and 19 after (a) radiation	one-year overall survival was 65% (d), 56% (a), and 40% (b)
Kropp et al. 2016 [155]	after	N	melanoma brain metastases	16 patients received various doses of radiation and ipilimumab after 15–150 weeks	one-year overall survival was 87%
Hiniker et al. 2016 [68]	concomitant	Y	metastatic melanoma, stage M1	20 patients received 18–50 Gy of radiation divided into fractions ranging from 2.5 to 25 Gy and concomitant ipilimumab injections	15% partial response, 15% complete remission
Qin et al. 2016 [144]	comparative	N	melanoma brain metastases	34 patients received 1–4 treatments of 6–20 Gy irradiation (median dose 16 Gy) and ipilimumab (h), 54 patients received 6–16 treatments of 2.5–3.5 Gy irradiation and ipilimumab (l)	median overall survival was 20 months (h) and 10 months (l), no differences in survival resulting from timing of treatments
Skrepnik et al. 2017 [157]	comparative	N	melanoma brain metastases	11 patients received ipilimumab and radiotherapy concurrently, 9 patients received ipilimumab after radiotherapy, 5 patients received ipilimumab before radiotherapy	no significant difference in survival
Patel et al. 2017 [167]	comparative	N	melanoma brain metastases	54 patients received 15–21 Gy of radiation, of which 34 received radiation alone (r), 7 received radiation and ipilimumab within 14 days (d), 14 received radiation and ipilimumab after more than 14 days (a)	one-year overall survival was 39% (r), 34% (a), and 42% (d)
Cohen-Inbar et al. 2017 [166]	comparative	N	melanoma brain metastases	32 patients received ipilimumab before or during radiation (d), 14 patients received ipilimumab after radiation (a)	Local recurrence-free duration was 19.6 months (d) and 3 months (a)
Schmidberger et al. 2018 [165]	comparative	N	melanoma brain metastases	27 patients received multiple doses of 2.5–3 Gy (h), 20 patients received ipilimumab before (b) and 21 after (a) differing types of radiotherapy	median overall survival was 9 months (b + a), 11 months (a), 3 months (b) and 3 months (h)

**Table 2:** Prospective clinical trials exploring ipilimumab with or without radiotherapy in metastatic melanoma.

Trial	Cohort Description	Treatment Groups	Progressive Disease	Stable Disease	Partial Response	Complete Response
Hodi et al. 2010 [122]	unresectable stage III or IV melanoma	320 patients received gp100 vaccine and ipilimumab (g + i), 109 patients received ipilimumab (i), 104 patients received gp100 (g)	(g + i) 75% (i) 64% (g) 86%	(g + i) 18% (i) 22% (g) 13%	(g + i) 7% (i) 12% (g) 2%	(g + i) 0.3% (i) 2% (g) 0%
Twyman-Saint Victor et al. 2014 [137]	metastatic melanoma, stage M1, mostly M1c (68%)	22 patients received 2–3 × 6–8 Gy targeted radiotherapy and ipilimumab 3–5 days after the last irradiation	64%	18%	18%	0%
Hiniker et al. 2016 [68]	metastatic melanoma, stage M1	20 patients received 18–50 Gy of radiation divided into fractions ranging from 2.5 to 25 Gy and concomitant ipilimumab injections	45%	25%	15%	15%

**Table 3:** Overview of clinical trials concerning combination of radiotherapy and anti-PD-1 treatment in various cranial metastatic settings. Clinical trials are sorted primarily by cancer and treatment setting, secondary criteria being chronological. Short descriptions of patient collective, treatment, and outcome are included.

Trial	Timing of Checkpoint Inhibition	Prospective (Y/N)	Cohort Description	Treatment	Outcome
Anti-PD-1, Brain Metastasis					
Ahmed et al. 2016 [158]	various	N	melanoma brain metastases	26 patients, receiving nivolumab during (73%) or after (27%) radiotherapy (84% single treatment, mostly 20–24 Gy, 16% fractionated treatment)	safety established, median overall survival of 11.8 months from radiotherapy
Aboudaram et al. 2017 [161]	various	N	melanoma brain metastases	17 patients received radiotherapy (r), 42 patients received radiotherapy and anti-PD-1 up to one month after radiotherapy (p)	Six-month progression-free survival was 65% (p) and 50% (r)
Nardin et al. 2018 [160]	various	N	melanoma brain metastases	25 patients, receiving durvalumab and various doses and fractions of irradiation	safety established, median overall survival of 14.6 months from radiotherapy
Trommer-Nestler et al. 2018 [143]	concomitant	N	melanoma brain metastases	13 patients received 18–22 Gy radiation and either pembrolizumab or nivolumab simultaneously (p), 13 patients received 18–20 Gy radiation (r)	after 6 months (p) 61% and (r) 15% of lesions regressed
Komatsu et al. 2018 [159]	various	N	melanoma brain metastases	5 patients receiving 10–13 3 Gy fractions of radiation and nivolumab 0–22 months after	partial response, stable disease and complete remission reported
Kotecha et al. 2019 [164]		N	various brain metastases	150 patients with 1003 brain metastases were treated with radiation and anti-PD-1, of these 367 metastases were treated within one half-life of anti-PD-1 (c), while 636 metastases were not (nc)	complete response was 50% (c) and 32% (nc); complete response was associated with overall survival; steroid treatment was detrimental

**Table 4:** Overview of clinical trials concerning comparison of anti-PD-1 and anti-CTLA-4 treatments in combination with radiotherapy in various cranial metastatic settings. Clinical trials are sorted primarily by cancer and treatment setting, secondary criteria being chronological. Short descriptions of patient collective, treatment, and outcome are included.

Trial	Timing of Checkpoint Inhibition	Prospective (Y/N)	Cohort Description	Treatment	Outcome
Anti-PD-1 Compared to Anti-CTLA-4, Brain Metastasis					
Qian et al. 2016 [163]	comparative	N	melanoma brain metastases	Patients received 12–24 Gy radiation, 32 patients received anti-CTLA-4 or anti-PD-1 concurrently (d) and 22 non-concurrently (a); in the same cohort 54 received anti-CTLA-4 (c) and 21 received anti-PD-1 (p)	median percent reduction in lesion volume at 1.5 months was 63% (d), 41% (n), 71% (p), and 48% (c)
Choong et al. 2017 [172]		N	melanoma brain metastases	26 patients received radiation (r), 28 received radiation and anti-CTLA-4 (c), 11 received radiation and anti-PD-1 (p), and 39 received radiation and v-Raf murine sarcoma viral oncogene homolog B1 (BRAF) and dual specificity mitogen-activated protein kinase kinase (MEK) inhibitors (b)	median overall survival was 11 months (r), 8 months (c), 20 months (p), and 18 months (b)
Gaudy-Marqueste et al. 2017 [173]		N	melanoma brain metastases	BRAF-mutated patients were treated with radiation (mr) (*n* = 29) or radiation combined with BRAF and/or MEK inhibitors alone (mm) (*n* = 34), combined with anti-CTLA-4 or anti-PD-1 (mc), or anti-CTLA-4 or anti-PD-1 alone (mi); BRAF wildtype patients were treated with radiation alone (r) or combined with anti-CTLA-4 (c) anti-PD-1 (p) or both (b)	two-year overall survival was 14% (mr), 9% (mm), 39% (mc), 54% (mi); one year overall survival was 14% (r), 41% (c), 64% (p), 75% (b)
Stokes et al. 2017 [162]	various	N, meta-analysis	melanoma brain metastases	1287 patients with melanoma brain metastases receiving radiation were analyzed, of which 185 also received anti-CTLA-4 or anti-PD-1/PD-L1 (c), and the rest receiving radiation only (r)	median overall survival was 11 months (c) and 6 months (r)
Anderson et al. 2017 [171]		N	melanoma brain metastases	23 patients received radiation and pembrolizumab (p), 31 patients received radiation and ipilimumab (i), 27 patients received radiation only (r)	complete response was 35% (p), 13% (i), and 4% (r)
Chen et al. 2018 [168]	comparative	N	melanoma, Non-small-cell lung carcinoma (NSCLC) and renal cancer (RCC) brain metastases	of NSCLC (*n* = 157), melanoma (*n* = 70), and RCC (*n* = 33) patients 69% received single or multiple 5–25 Gy fractions of radiation, with or without conventional therapy (r), 20% received non-concurrent (n) and 11% concurrent (c) anti-PD-1 or anti-CTLA-4 with radiation	median overall survival was 13 months (r), 15 months (n), and 25 months (c)
Robin et al. 2018 [169]	comparative	N	melanoma brain metastases	25 patients received radiation and anti-CTLA-4 within 8 weeks (i), 13 patients received radiation and anti-PD-1 with or without anti-CTLA-4 within 8 weeks (p)	median progression free survival was 2 months (i) and 23 months (p)
Lehrer et al. 2019 [170]	comparative	N, meta-analysis	melanoma brain metastases	218 patients across 7 studies received radiation and checkpoint inhibitors concurrently (c) before (b) or after (a) radiation	one-year overall survival was 65% (c), 41% (b), and 56% (a)
Minniti et al. 2019 [145]	concomitant	N	melanoma brain metastases	45 patients received radiation and ipilimumab (i), 35 patients received radiation and nivolumab (n)	median overall survival was 22 months (n) and 15 months (i)

**Table 5:** Overview of clinical trials concerning combination of radiotherapy and anti-PD-1 or anti-CTLA-4 treatment in NSCLC and other thoracic malignancies. Clinical trials are sorted primarily by cancer and treatment setting, secondary criteria being chronological. Short descriptions of patient collective, treatment, and outcome are included.

Trial	Timing of Checkpoint Inhibition	Prospective (Y/N)	Cohort Description	Treatment	Outcome
anti-PD-1 or anti-CTLA-4, NSCLC					
Antonia et al. 2017 [176]	after	Y	stage III NSCLC	all patients received chemoradiotherapy (platin based), 473 of which received durvalumab within at least 42 days (d), while 236 patients received placebo after chemoradiotherapy (p), of	median progression-free survival from randomization was 17 months (d) and 6 months (p)
Shaverdian et al. 2017 [177]	after	Y	NSCLC	97 patients receiving pembrolizumab, 42 patients had previously received radiotherapy (r) and 55 had not (n)	median progression-free survival was 4 months (r) and 2 months (n)
von Reibnitz et al. 2018 [175]	various	N	various thoracic tumors/metastases	62 patients received radiation and anti-PD-1/PD-L1, 12 patients received anti-CTLA-4 and radiation, 5 patients received both anti-PD-1/PD-L1 and anti-CTLA-4	no differences among groups
Lesueur et al. 2018 [180]	comparative	N	metastatic NSCLC	104 patients received radiation and nivolumab with varying intervals	one-year overall survival was 48%, no correlation with nivolumab timing
Foster et al. 2019 [178]	various	N, meta-analysis	NSCLC	44,498 patients were analyzed	stereotactic radiotherapy and checkpoint inhibition predicted superior survival, independent on their combination
Shepard et al. 2019 [179]	various	N	metastatic NSCLC	34 patients received radiation, 12 patients received radiation and anti-PD-1/PD-L1	no differences among groups
Yamaguchi et al. 2019 [181]	before	N	NSCLC, stage III or IV	66 patients received radiation before nivolumab treatment (r), 58 patients received nivolumab without radiation (n)	median progression-free survival was 204 days (r) and 79 days (n), median overall survival was 562 days (r) and 524 days (n)

**Table 6:** Overview of clinical trials concerning combination radiotherapy and immunotherapy in various malignancies. Clinical trials are sorted primarily by cancer and treatment setting, secondary criteria being chronological. Short descriptions of patient collective, treatment, and outcome are included.

Trial	Timing of Checkpoint Inhibition	Prospective (Y/N)	Cohort Description	Treatment	Outcome
Various Malignancies					
Kwon et al. 2014 [187]	after	Y	bone metastasis from castration-resistant prostate cancer	400 patients received 8 Gy of radiation (r), 399 patients received 8 Gy of radiation followed by ipilimumab up to two days later (i)	median overall survival was 10 months (r) and 11 months (i), not statistically significant
Tang et al. 2017 [147]	various	Y	various malignancies	35 patients with various malignancies received either 12.5 Gy of radiation 4 times or 6 Gy radiation 10 times, combined with ipilimumab either concomitantly or sequentially	combination was safe, limited value due to the small and varied cohort
Qin et al. 2018 [183]	various	N	treatment resistant Hodgkin’s lymphoma	three patients were treated, two with radiation and concomitant nivolumab and one with radiation and nivolumab 2 months later	all patients alive and in complete remission after 23–27 months (historical complete remission rate under anti-PD-1: 17–22%)
Quéro et al. 2019 [184]		N	treatment resistant Hodgkin’s lymphoma	four patients were treated with radiation and anti-PD-1	after median follow-up of 13-months, all patients alive with complete metabolic response
Floudas et al. 2019 [182]		Y	metastatic colorectal cancer	10 patients received PD-1 blocking protein AMP-224 on the last day of 1–3 radiation treatments, delivering 9 Gy each and low-dose cyclophosphamide	no objective response was noted, median overall survival was 6 months
Liu et al. 2019 [186]		Y	Pretreated, microsatellite stable cholangiocarcinoma	3 patients received PD-1 blockade and stereotactic radiotherapy delivering 11–13 Gy in 4–5 fractions	2 patients achieved partial response; one patient achieved complete response maintained for 11 months
